# *CLEC7A* as a potential biomarker of atherosclerotic response to aspirin therapy: a multi-omics analysis

**DOI:** 10.3389/fimmu.2026.1727020

**Published:** 2026-07-29

**Authors:** Ke Cai, Dongjie Chen, Jingyu Wang, Jing Bai, Mengting Wang, Jianming Guo, Feng Liu, Jin-ao Duan, Shulan Su

**Affiliations:** 1 Jiangsu Collaborative Innovation Center of Chinese Medicinal Resources Industrialization, National and Local Collaborative Engineering Center of Chinese Medicinal Resources Industrialization and Formulae Innovative Medicine, and Jiangsu Provincial Key Laboratory of Functional Substances in Traditional Chinese Medicine Formulae and Innovative Drug Discovery, Nanjing, China; 2 Shaanxi Institute of International Trade and Commerce, Xianyang, China; 3 State Key Laboratory of Technologies for Chinese Medicine Pharmaceutical Process Control and Intelligent Manufacture (Jiangsu Kanion Pharmaceutical Co., Ltd. & Nanjing University of Chinese Medicine), Jiangsu, Nanjing, China

**Keywords:** aspirin non-responsiveness, atherosclerosis, biomarker, CLEC7A, multi-omics analysis

## Abstract

Many patients with atherosclerotic cardiovascular disease (ASCVD) do not respond well to aspirin therapy. This study employed a multi-omics approach to identify key genes associated with aspirin non-responsiveness, thereby providing potential molecular targets and theoretical basis for improving the precision treatment strategies for ASCVD patients. One aspirin non-responsiveness dataset and two atherosclerosis-related datasets were obtained from the Gene Expression Omnibus database. After identifying differentially expressed genes through weighted gene co-expression network analysis and differential expression analysis, we used machine learning techniques to screen for key genes and establish predictive models. The results showed that two key genes (*CLEC7A* and *RGS1*) performed well in diagnosing aspirin non-responsiveness and ASCVD (area under the curve = 0.701–0.986). Among them, *CLEC7A* was significantly overexpressed in the aspirin non-responsiveness and ASCVD datasets (*P* < 0.0001) and was identified as a potential risk factor. Furthermore, ELISA results showed overexpression of CLEC7A in the aortic tissue and serum of ASCVD mice (*P* < 0.0001). In addition, immune infiltration analysis suggested that aspirin non-responsiveness mediated by CLEC7A may involve natural killer cells (*P* = 0.04). However, CLEC7A-mediated ASCVD may involve regulatory T cells and neutrophils (*P* < 0.0001), a finding further supported by immunofluorescence co-localization analyses. We constructed a regulatory network for CLEC7A and conducted small-molecule virtual screening for *CLEC7A*. In summary, *CLEC7A* can serve as a potential biomarker to identify ASCVD patients with a low aspirin response.

## 1 Introduction

Atherosclerosis is the underlying cause in most cases of myocardial infarction, coronary heart disease, and disabling peripheral artery disease (1). It has a high incidence and mortality and ranks first regarding disease burden (2). Due to its chronic and persistent nature, atherosclerotic cardiovascular disease (ASCVD) markedly reduces patients’ quality of life and represents a major public health problem. A critical limitation in preventive management is non-responsiveness to aspirin, in which an inadequate antiplatelet effect compromises treatment efficacy in a substantial subset of patients (3).

Aspirin is a cornerstone antiplatelet agent for ASCVD. Its proven benefit in secondary prevention stems from the irreversible inhibition of platelet cyclooxygenase-1 (COX-1), which suppresses thromboxane A2 (TXA_2_) production and platelet aggregation (4, 5). A major therapeutic limitation of aspirin therapy is the marked interindividual variability in response. This was commonly referred to as “aspirin resistance” in earlier literature (6); however, it is more accurately described as “aspirin non-responsiveness” to reflect the continuum of response and multifactorial etiology (7, 8).

Therefore, the ability to prospectively identify patients who are aspirin non-responsive is clinically important. Biomarkers that predict a low antiplatelet response would enable personalized therapy by redirecting non-responders to alternative strategies, thus improving patient outcomes. Investigating the genetic and molecular basis of aspirin non-responsiveness is essential to identify such biomarkers.

We analyzed a prospective cohort of patients with aspirin non-responsiveness and ASCVD. Using an integrated bioinformatics approach incorporating weighted gene co-expression network analysis (WGCNA), machine learning techniques, single-cell transcriptomic analysis, virtual screening, and protein docking—combined with validation by ELISA and immunofluorescence—we aimed to identify key genes associated with aspirin non-responsiveness in the prevention and treatment of atherosclerosis. These findings may enable patient stratification based on predicted treatment response and support personalized antiplatelet therapy to improve ASCVD prevention and management.

## 2 Materials and methods

### 2.1 Data collection and preparation

The datasets utilized in this study were sourced from the Gene Expression Omnibus (GEO) database (https://www.ncbi.nlm.nih.gov/geo/) (**Table 1**). Specifically, GSE38511 comprised microarray data from peripheral blood samples of 8 aspirin-resistant and 9 aspirin-sensitive patients (9, 10). GSE28829 contained microarray data from plaque tissue samples of 16 patients with advanced atherosclerosis and 13 patients with early atherosclerosis (11). Additionally, the GSE43292 dataset included samples from a total of 64 patients with hypertension. Of these, 32 patients provided carotid plaque tissues (case group), while the other 32 patients provided corresponding macroscopically intact arterial tissues (control group) (12).

**Table 1:** Details of the gene expression omnibus (GEO) datasets.

Dataset	Platform	Number of samples (groups)
GSE38511	GPL570 [HG-U133_Plus_2] Affymetrix Human Genome U133 Plus 2.0 Array	17 (8 aspirin-resistant, 9 aspirin-sensitive)
GSE28829	GPL570 [HG-U133_Plus_2] Affymetrix Human Genome U133 Plus 2.0 Array	29 (16 advanced atherosclerosis, 13 early atherosclerosis)
GSE43292	GPL6244 [HuGene-1_0-st] Affymetrix Human Gene 1.0 ST Array [transcript (gene) version]	64 (32 carotid plaque tissue, 32 macroscopically intact arterial tissue)

GEO, Gene Expression Omnibus.

### 2.2 Differential gene expression analysis

Data preparation for individual diseases involved comparing ASCVD and aspirin non-responsiveness datasets using the Linear Models for Microarray (LIMMA) package in R (version 4.4.3). The datasets were batch-corrected using the combat function in the “sva” package in R. Differentially expressed genes (DEGs) were identified for each disease group and compared to the control group. DEGs for ASCVD were defined by a *P* value <0.05 and |log2FC|>0.15, while for aspirin non-responsiveness, DEGs were identified with an adjusted *P* value of 0.05 and |log2FC|>0.5 (a stricter threshold chosen to account for the dataset’s greater variability and smaller sample size). Subsequently, the “ggplot2” and “pheatmap” packages in R were used to visualize the expression patterns of DEGs as volcano maps and heatmaps, respectively, with blue indicating low expression and red indicating high expression.

### 2.3 WGCNA analysis

We conducted an analysis using the R package “WGCNA” to identify gene modules significantly linked to disease clinical features (13). Initially, we employed hierarchical cluster analysis to eliminate outlier samples. Subsequently, the “pickSoftThreshold” function in the WGCNA package was used to determine the optimal soft threshold *β*. This step produced a neighbor-joining matrix, which was transformed into a topological overlap matrix (TOM). Genes with comparable expression profiles were grouped into distinct modules using average linkage hierarchical clustering based on diverse TOM metrics. Lastly, we assessed the associations between these modules and clinical features through correlation analyses.

### 2.4 Identification of shared genes and functional enrichment analysis

By integrating DEGs with module genes identified through WGCNA, we successfully pinpointed common key genes implicated in the pathogenesis of both ASCVD and aspirin non-responsiveness. To elucidate the biological functions and signaling pathways of these genes, we employed the “clusterProfiler” package to conduct Gene Ontology (GO) and Kyoto Encyclopedia of Genes and Genomes (KEGG) pathway enrichment analyses, setting *P* < 0.05 as the threshold (14).

### 2.5 Protein–protein interaction network analysis

The STRING database (https://string-db.org/) was used to investigate PPI networks of the identified genes, with a minimum required interaction score set to medium confidence (0.4), and the networks were visualized in Cytoscape 3.10.3. Subsequently, module analysis of the PPI network was performed using the MCODE plugin. Hub genes were identified using the cytoHubba plugin, including MNC (Neighborhood Component Centrality), MCC (Maximum Clique Centrality), Degree, and EPC (Edge Percolated Component). For each algorithm, the top 20 highest-ranked genes were selected. The intersection of these four lists yielded a final set of high-confidence hub genes (15). Furthermore, the GeneMANIA database (https://genemania.org/) was used to analyze the gene network of the hub genes (16).

### 2.6 Machine learning and key gene analysis

Machine learning represents an innovative approach for analyzing algorithms to identify pivotal therapeutic resistance genes associated with aspirin. The Least Absolute Shrinkage and Selection Operator (LASSO) is a regularized regression algorithm implemented in the “glmnet” package (17). Additionally, the Random Forest (RF) algorithm was executed utilizing the “randomForest” package in the R programming environment (18). Following that, Support Vector Machine-Recursive Feature Elimination (SVM-RFE) was used to eliminate recursive features. Ten-fold cross-validation was applied during LASSO feature selection to determine the optimal λ value. RF importance scores and SVM-RFE cross-validation errors were used to reduce model instability and minimize overfitting. The predictive accuracy of key gene identification is evaluated using Receiver Operating Characteristic (ROC) curves.

### 2.7 Immune infiltration and single-cell analysis

The study employed the CIBERSORT tool, which uses a gene-expression-based deconvolution algorithm, to estimate the proportions of 22 infiltrating lymphocyte subtypes in each sample group. Visualization and correlation analyses were conducted using the “ggplot2” R package. A subsequent Pearson correlation analysis was performed to explore the relationship between key genes and immune cell subtypes, with a significance threshold of *P* < 0.05 (19). Gene Set Enrichment Analysis (GSEA) was carried out utilizing the clusterProfiler package with the c2.cp.kegg_medicus.v2025.1.Hs.symbols.gmt gene set from the Molecular Signature Database (MSigDB), applying a significance threshold of *P* < 0.05. The GSEA results were visualized using the “ggplot2” package (20).

### 2.8 Constructing molecular regulatory networks of key genes

Initially, mRNA–miRNA interactions were investigated using online RNA interaction prediction tools, including TargetScan, miRDB, microT, and miRWalk. The merged data were taken as intersections, and interactions were deemed reliable only if they were consistently predicted by all tools. Subsequently, miRNA–lncRNA interactions were examined using databases such as miRNet and lncbasev3, with merged data taken as intersections to confirm their consistency in identification.

### 2.9 Ligand-based virtual screening

The AlphaFold structure of the CLEC7A protein (AlphaFold ID: AF-Q9BXN2-F1) was submitted to the Proteins Plus Server (https://proteins.plus/) web server for online prediction of protein sites. Subsequently, virtual screening was performed using the Enamine REAL databases (containing more than 4.6 million drug-like molecules) provided by DrugCLIP (https://www.drugclip.com), an artificial intelligence-driven ultra-high-throughput virtual drug screening platform (21). Finally, Discovery Studio was used to visualize docking results.

### 2.10 Establishment of the ASCVD animal model and sample collection

The experimental protocol was approved by the Animal Experiment Ethics Committee of Nanjing University of Traditional Chinese Medicine (Ethics Number: 202501A006). Eight male C57BL/6 mice and 16 ApoE^-/-^ mice (6–8 weeks old) on a C57BL/6 background (animal license no. SCXK (Shanghai) 2024-0001, ethical license no. 110324251100821081) were purchased from SPF (Beijing Biotechnology Co., Ltd.), and housed in the Laboratory Animal Center of Nanjing University of Chinese Medicine. The temperature was controlled at 22 ± 2°C, the relative humidity at 55 ± 10%, and a 12-h light–dark cycle was used, with food and water provided ad libitum.

After 1 week of adaptive feeding, ApoE^-/-^ mice were randomly divided into two groups: the ASCVD group and the aspirin (ASA) group. These mice were fed a high cholesterol diet (#D12079B, SPF, Beijing, China) for 18 weeks to establish an atherosclerosis mouse model (22). C57BL/6 mice were fed a basic diet for 18 weeks as the control group. Starting in the 11th week, aspirin (Push Biotechnology, China) was administered at a dose of 12.7 mg·kg^-1^·d^-1^ *via* gavage in the ASA group, while the ASCVD group and the control group received an equal volume of saline for 8 weeks. After reaching the specified experimental endpoint, the mice were fasted for 12 h, anesthetized with 2–5% isoflurane before tissue collection, and euthanized by cervical dislocation to ensure they were unconscious before death. Animal welfare was strictly monitored throughout the process. Blood was collected through the supraorbital sinus, and serum was collected by centrifuging at 1000 × g at 4 °C for 15 min. The serum and aortic tissues were stored at –80 °C for subsequent analyses. The remaining aortic tissue was fixed with 4% paraformaldehyde for subsequent pathological sectioning and staining.

### 2.11 Oily red staining of the aortic tissue

Aortic tissues were embedded in optimal cutting temperature compound (OTC) and sectioned with the cryostat. The slices were baked at 60°C for 30 min, washed with 70% absolute alcohol, and stained with oil red O for 10 min. They were then washed with 70% absolute alcohol, washed with distilled water, and stained with hematoxylin for 2 min. After removal of excess staining solution, the sections were dehydrated, cleared in dimethylbenzene, and mounted with neutral gum. Stained sections were examined under a light microscope.

### 2.12 Enzyme-linked immunosorbent assay

The protein concentration of CLEC7A was analyzed in the aortic tissue and serum samples collected from the mice. The aortic tissue was homogenized in ice-cold RIPA lysis buffer containing 1% protease inhibitor mixture using a mechanical homogenizer. The homogenate was centrifuged at 12,000 × g and 4 °C for 15 min, and the supernatant was collected for analysis. The bicinchoninic acid (BCA) protein assay kit was used to determine the protein concentration of the tissue lysates to ensure equal sample loading. Subsequently, we quantified CLEC7A protein concentrations in aortic tissue and serum samples from mice using a substrate-based ELISA kit (Dreambio, China) according to the manufacturer’s protocols.

### 2.13 Immunofluorescence staining

The aortic heart tissue was fixed in 4% paraformaldehyde, embedded in OCT, and cut into 5 µm sections. The sections were permeabilized with 0.1% Triton X-100 (Beyotime Biotechnology, P0096) for 20 min, and then blocked with 5% bovine serum albumin (BSA) at 37 °C for 1 h. After three washes with phosphate-buffered saline (PBS), the sections were incubated overnight at 4 °C with the following primary antibodies: anti-Foxp3 (#YM8133, immunoway, Suzhou, China), anti-CLEC7A (#YM9238, immunoway, Suzhou, China), and anti-Ly6g (#YM8307, immunoway, Suzhou, China). On the second day, the sections were incubated with the corresponding fluorescently labeled secondary antibodies for 1 h, followed by counterstaining with DAPI for the nuclei. Fluorescence microscopy (Zeiss, Germany) was used to capture and measure fluorescence intensity.

For statistical analysis, three regions were selected for quantitative histological measurements, and the mean values were calculated. In addition, continuous sections were chosen, and consistent fields of view with different markers were recorded. Under ×400 magnification, the average optical density of Foxp3, CLEC7A, and Ly6g levels were measured in three randomly selected non-overlapping microscopic fields using Image J software.

### 2.14 Statistical analysis

Statistical analyses were performed using R software (version 4.4.3) and GraphPad Prism 8.0.2 (GraphPad Software Inc., San Diego, CA, USA). Data normality was assessed using the Shapiro–Wilk test. For comparisons among multiple groups, one-way analysis of variance (ANOVA) followed by Tukey’s multiple-comparison test was performed. Statistical significance was defined as a *P* < 0.05.

## 3 Results

### 3.1 Screening of aspirin non-responsiveness and ASCVD DEGs

**Figure 1:**
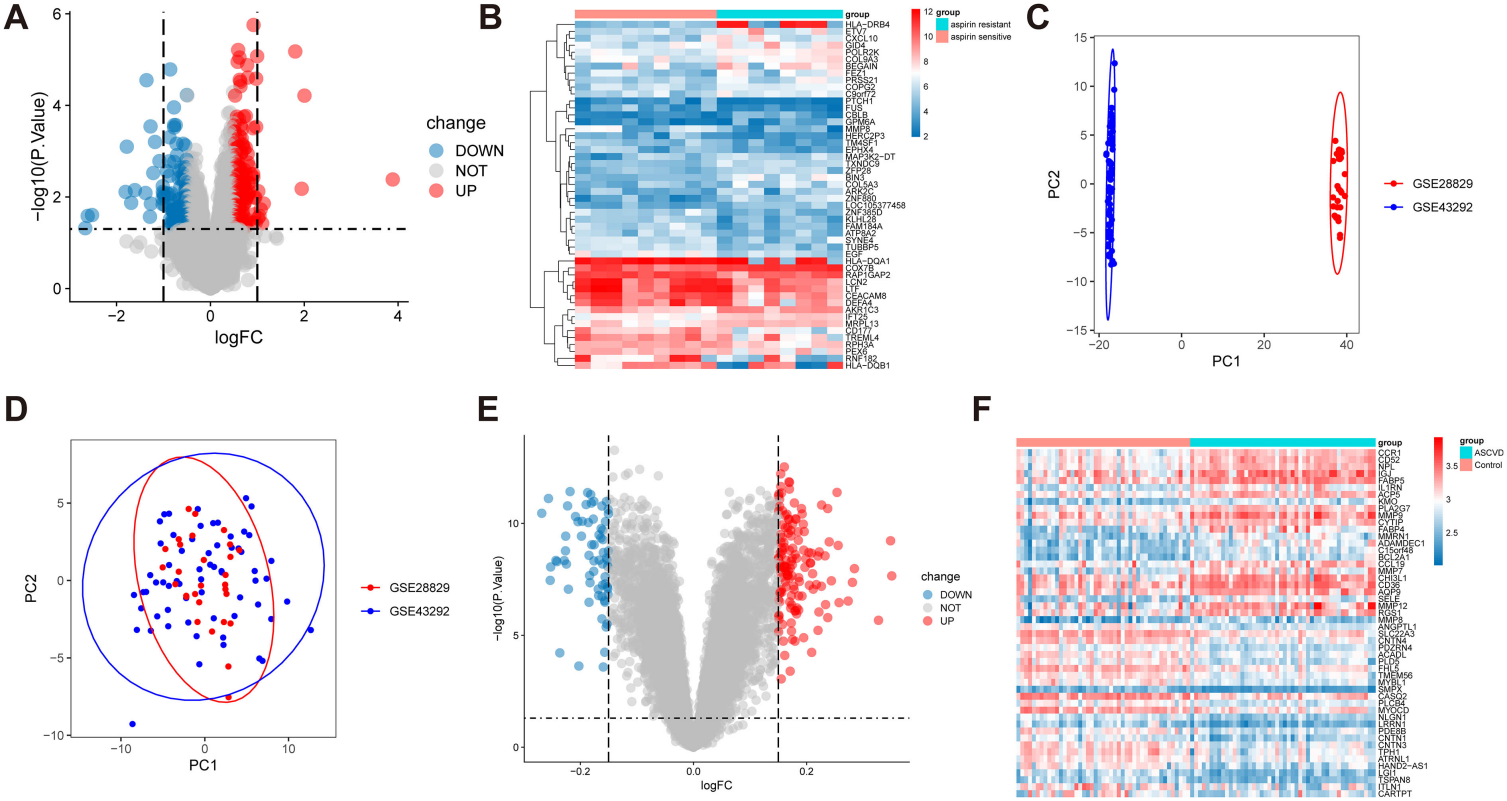
Removal of batch effects and identification of DEGs in the AR and ASCVD datasets. **(A, B)** DEG heatmap and volcano plot in the AR group. **(C, D)** PCA plots show the expression patterns in the two ASCVD datasets before and after batch-effect removal. **(E, F)** DEG heatmap and volcano plot in the ASCVD group. AR, Aspirin resistant; ASCVD, Atherosclerotic cardiovascular disease; DEG, differentially expressed gene; PCA, Principal-component analysis.

The aspirin non-responsiveness-related dataset GSE38511 was normalized initially, followed by the generation of a volcano plot (**Figure 1A**). Subsequently, the LIMMA package was used to identify 314 DEGs (*P* < 0.05, |log2 FC|>0.5), comprising 180 upregulated genes and 134 downregulated genes. Heatmaps displaying the top 25 DEGs for both up- and downregulated genes are illustrated in **Figure 1B**. Regarding the ASCVD-related datasets GSE28829 and GSE43292, an assessment of batch effects revealed a significant impact between the datasets (**Figure 1C**). By employing the “sva” package, the batch effect for ASCVD was effectively mitigated (**Figure 1D**) to ensure the reliability of subsequent analyses. The LIMMA package was then applied to identify 206 DEGs (*P* < 0.05, |log2 FC|>0.15), with 139 upregulated genes and 67 downregulated genes. Volcano plots depicting all DEGs associated with ASCVD are presented in **Figure 1E**, while heatmaps displaying the top 25 DEGs for both up and downregulated genes are shown in **Figure 1F**.

### 3.2 Screening for key modules by WGCNA

**Figure 2:**
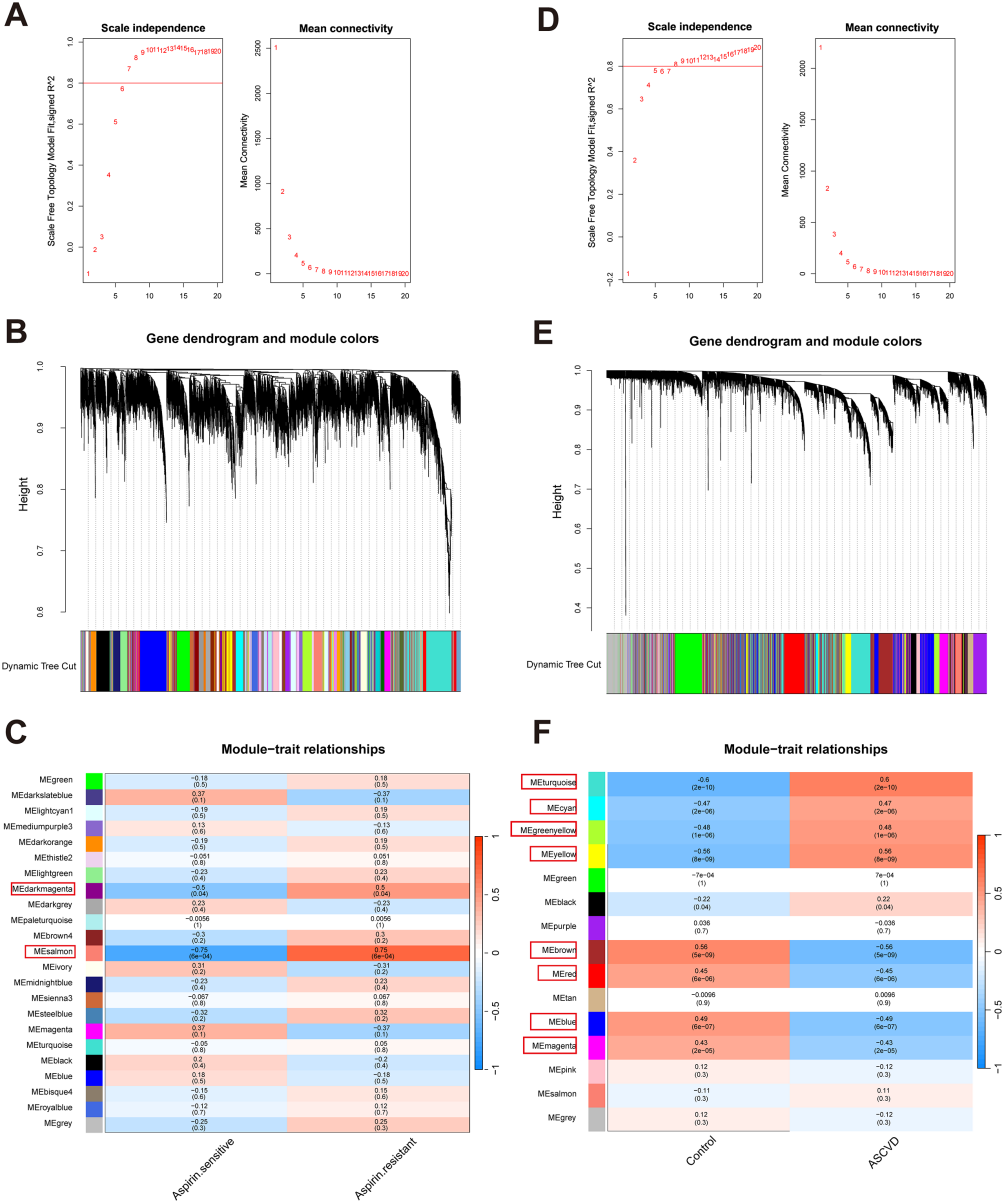
WGCNA of the AR and ASCVD datasets. **(A)** Determination of soft-threshold power for AR. **(B)** Cluster dendrogram of AR highly connected genes in key modules. **(C)** Relationships between modules and traits in AR. Correlations and *P* values are included in each cell. **(D)** Calculation of soft-threshold power for ASCVD. **(E)** Cluster dendrogram of ASCVD modules with highly connected genes. **(F)** Module–trait relationships in ASCVD. A correlation and *P* value are included in each cell. AR, Aspirin resistant; ASCVD, Atherosclerotic cardiovascular disease; WGCNA, Weighted gene co-expression network analysis.

To investigate the relationship between diseases and key genes, we employed WGCNA and differential expression analysis. In the aspirin non-responsiveness group, a fit index above 0.8 confirmed scale-free topology, with a *β* value of 7 (**Figure 2A**). This led to the construction of an adjacency matrix and hierarchical clustering based on the TOM dissimilarity measure (**Figure 2B**). We identified 23 co-expression modules, with those showing *P* < 0.05 deemed significant. Notably, the darkmagenta and salmon modules, comprising 461 genes, were significantly correlated with aspirin non-responsiveness (**Figure 2C**). In the ASCVD group, WGCNA identified an optimal *β* value of 8 (**Figure 2D**), yielding 15 modules. The turquoise, cyan, green-yellow, and yellow modules showed strong positive correlations, whereas the brown, red, blue, and magenta modules showed strong negative correlations with ASCVD, involving 5904 genes (**Figures 2E, F**). Genes in these key modules could serve as candidate cell-type-specific markers.

### 3.3 Analysis of the shared genes and functional enrichment

**Figure 3:**
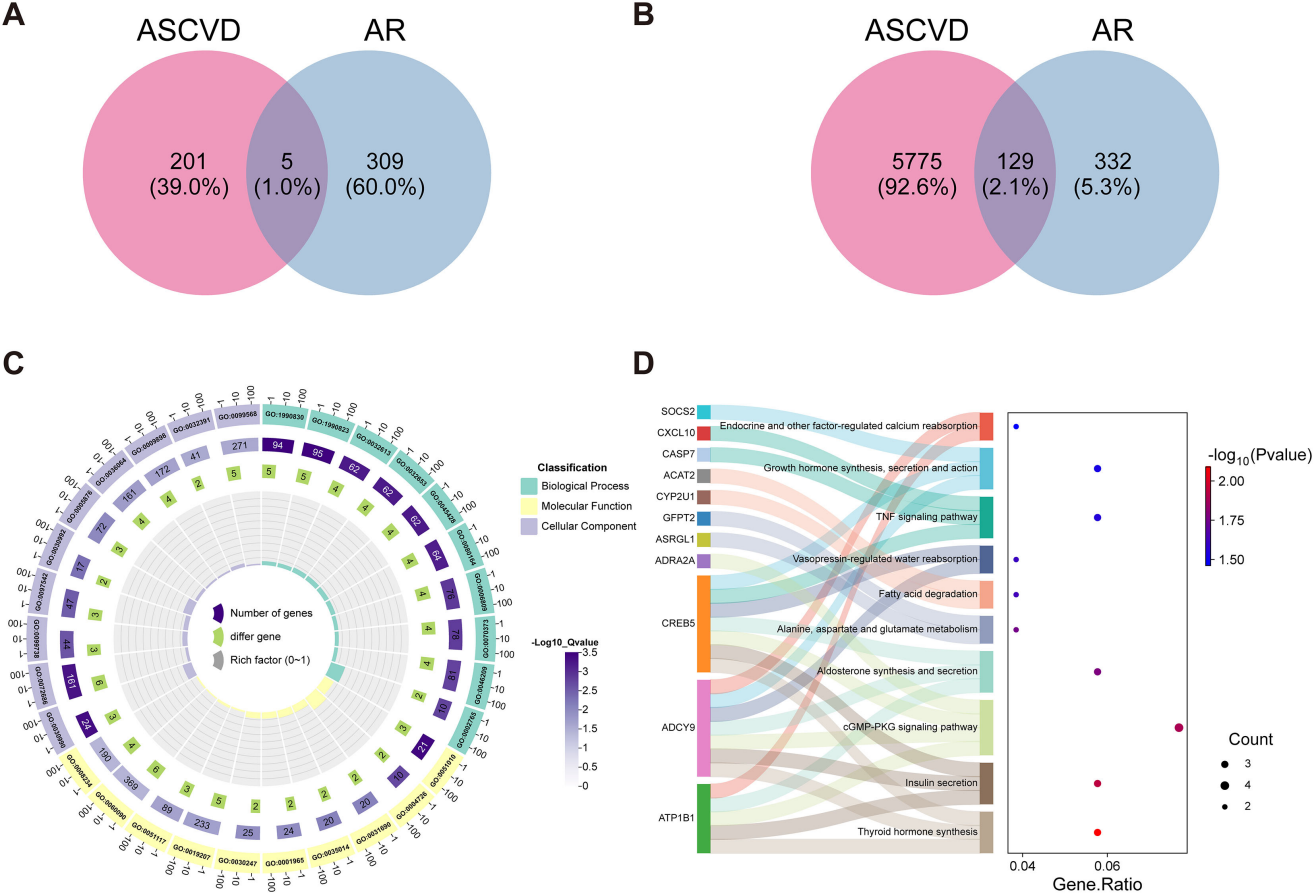
Shared gene signatures and functional enrichment between AR and ASCVD. **(A)** The shared DEGs between AR and ASCVD are obtained by overlapping their DEGs of them. **(B)** The shared genes between the WGCNA modules for AR and ASCV were identified by overlapping the modules. **(C, D)** Shared genes were represented by bar plots displaying GO and KEGG enrichment. AR, Aspirin resistant; ASCVD, Atherosclerotic cardiovascular disease; DEG, differentially expressed gene; GO, Gene Ontology; KEGG, Kyoto Encyclopedia of Genes and Genomes; WGCNA, Weighted gene co-expression network analysis.

To investigate the co-pathogenesis of aspirin non-responsiveness and ASCVD, we intersected the previously identified DEGs with genes identified through WGCNA. This intersection revealed five overlapping genes between aspirin non-responsiveness and ASCVD DEGs (**Figure 3A**) and 129 overlapping genes from the WGCNA analysis (**Figure 3B**), totaling 134 genes potentially implicated in both conditions. Functional annotation and enrichment analysis of these genes (**Figures 3C, D**) aimed to uncover potential biological changes linking aspirin non-responsiveness and ASCVD. GO analysis showed these genes were significantly involved in pathways related to endothelial damage, immune activation, fiber repair, and plaque stability (**Supplementary Table 1**). Notably, enriched GO terms included cellular response to leukemia inhibitory factor (GO:1990830), interleukin (IL)-10 production (GO:0032653), and regulation of nitric oxide (NO) biosynthesis (GO:0006809), indicating that immune cell activation and macrophage inflammatory factor release may significantly contribute to aspirin non-responsiveness and co-pathogenesis of ASCVD. KEGG enrichment supported these findings, highlighting pathways related to vascular inflammation, endothelial function, and plaque stability, such as the cyclic guanosine monophosphate–protein kinase G (cGMP–PKG) and tumor necrosis factor (TNF) signaling pathways (**Supplementary Table 2**).

### 3.4 PPI network and hub genes analysis

**Figure 4:**
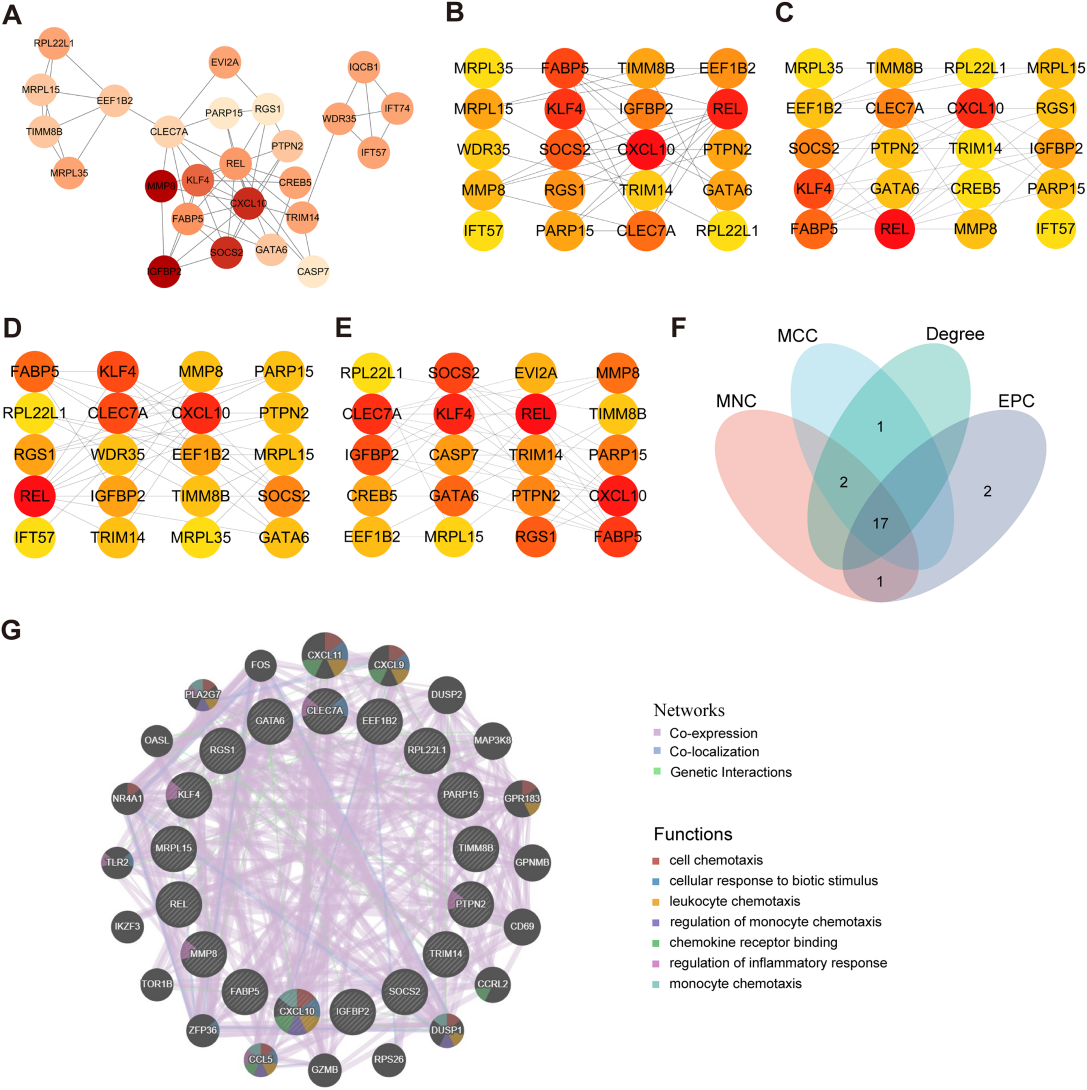
PPI network and Hub genes analysis. **(A)** The first module of the PPI network. **(B–E)** Four algorithms (MNC, MCC, Degree, EPC) from the cytoHubba plugin were used to identify the top 20 hub genes. **(F)** Seventeen common hub genes were identified by four algorithms in the cytoHubba plugin. **(G)** Gene networks were further constructed using GeneMANIA. MNC, Neighborhood Component Centrality; MCC, Maximum Clique Centrality; EPC, Edge Percolated Component; PPI, Protein–protein interaction.

Genes rarely function in isolation. They often encode proteins that interact with one another. To investigate these protein interactions, we constructed a PPI network using shared genes identified via the STRING tool. This network included 122 nodes and 327 edges. The MCODE plugin was utilized to identify the most densely connected module, comprising 25 nodes and 62 edges (**Figure 4A**). Additionally, we employed four cytoHubba algorithms to determine the top 20 hub genes (**Figures 4B–E**). Through comprehensive bioinformatics analysis, 17 common hub genes were identified: *REL, CXCL10, KLF4, FABP5, CLEC7A, SOCS2, IGFBP2, EEF1B2, RGS1, PARP15, GATA6, MRPL15, TIMM8B, MMP8, PTPN2, TRIM14*, and *RPL22L1* (**Figure 4F**; **Table 2**). Further gene networks were constructed using GeneMANIA, revealing that these hub genes are linked to innate immune and inflammatory responses, particularly in leukocyte-mediated cytotoxicity, inflammatory regulation, and chemokine production (**Figure 4G**).

**Table 2:** The hub genes.

Gene symbol	Gene name	logFC	Score of degree	Score of EPC	Score of MCC	Score of MNC
*REL*	REL Proto-Oncogene	0.334	13	15.883	96	13
*CXCL10*	C-X-C Motif Chemokine Ligand 10	1.046	12	15.830	110	12
*KLF4*	KLF Transcription Factor 4	0.235	8	15.592	84	8
*FABP5*	Fatty Acid Binding Protein 5	0.404	7	15.307	78	7
*CLEC7A*	C-Type Lectin Domain Containing 7A	0.801	8	15.535	38	6
*SOCS2*	Suppressor Of Cytokine Signaling 2	-0.099	6	15.055	54	6
*IGFBP2*	Insulin-Like Growth Factor Binding Protein 2	-0.336	5	14.777	26	5
*EEF1B2*	Eukaryotic Translation Elongation Factor 1 Beta 2	0.182	5	9.482	13	4
*RGS1*	Regulator Of G Protein Signaling 1	0.637	5	14.221	13	4
*PARP15*	Poly (ADP-Ribose) Polymerase Family Member 15	0.651	4	13.891	12	4
*GATA6*	GATA Binding Protein 6	-0.146	4	14.031	12	4
*MRPL15*	Mitochondrial Ribosomal Protein L15	0.333	4	7.702	12	4
*TIMM8B*	Translocase Of Inner Mitochondrial Membrane 8 Homolog B	0.438	4	7.967	12	4
*MMP8*	Matrix Metallopeptidase 8	-1.807	4	13.909	8	4
*PTPN2*	Protein Tyrosine Phosphatase Non-Receptor Type 2	0.349	4	13.864	12	4
*TRIM14*	Tripartite Motif Containing 14	0.200	4	12.971	7	3
*RPL22L1*	Ribosomal Protein L22 Like 1	0.378	3	7.363	6	3

### 3.5 Identification and validation of the key genes of aspirin non-responsiveness

**Figure 5:**
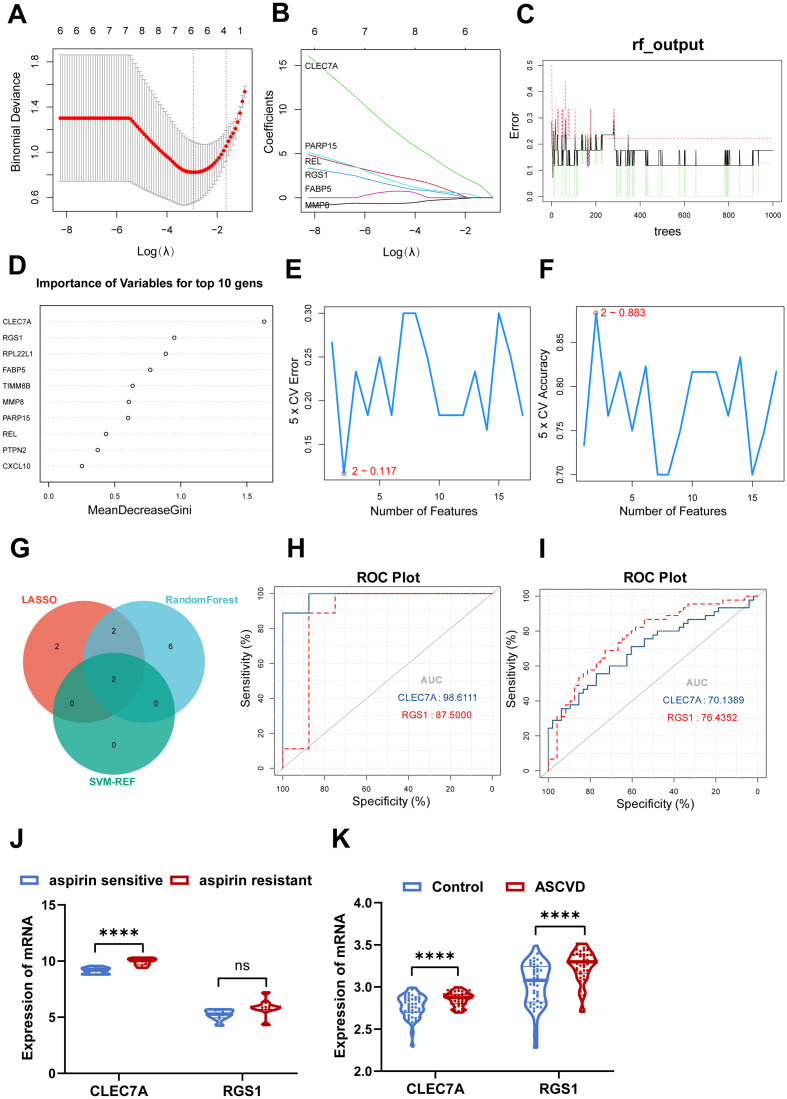
Identification of the key genes of aspirin non-responsiveness. **(A, B)** Six key genes were identified from the hub genes by machine learning LASSO regression. **(C, D)** Ten key genes were also identified from the hub genes by the Random Forest (RF) algorithm. **(E, F)** Two key genes were also identified from the hub genes by the SVM-RFE algorithm. **(G)** Two key genes were identified by overlapping *CLEC7A* and *RGS1*. **(H, I)** ROC curves of *CLEC7A* and *RGS1* in the training group for AR and ASCVD. **(J, K)** Differential expression of *CLEC7A* and *RGS1* in the training group for AR and ASCVD. AR, Aspirin resistant; ASCVD, Atherosclerotic cardiovascular disease. Data were shown as mean ± SD (ns *P* > 0.05, ****P > 0.0001).

To identify key diagnostic gene targets distinguishing aspirin-resistant (AR) from aspirin-sensitive (AS) groups, three algorithms (LASSO, Random Forest, and SVM-RFE) were applied using the 17 shared genes. LASSO regression (λ = 0.05254633) identified six potential biomarkers (**Figures 5A, B**). The Random Forest classifier ranked the top 10 genes by importance (**Figures 5C, D**). The SVM-RFE algorithm pinpointed two genes with optimal CV error and accuracy (**Figures 5E, F**). By intersecting results from these algorithms, we identified two common biomarkers for aspirin non-responsiveness, *CLEC7A* and *RGS1* (**Figure 5G**). ROC analysis was then conducted to evaluate the specificity and sensitivity of these genes for diagnosing aspirin non-responsiveness and ASCVD. Interestingly, for aspirin non-responsiveness biomarkers, good results were obtained for the two genes *CLEC7A* (area under the curve [AUC] = 0.986) and *RGS1* (AUC = 0.875) (**Figure 5H**). The same ROC analysis was performed in the ASCVD group. The predictive performance of each biomarker was stable: *CLEC7A* (AUC = 0.701) and *RGS1* (AUC = 0.764) (**Figure 5I**). We analyzed the aspirin non-responsiveness and ASCVD expression levels of the two discovery cohorts. Compared to the AS group, the expression level of *CLEC7A* was significantly elevated in the AR group (*P* < 0.0001), while *RGS1* showed no significant difference (*P*>0.05) (**Figure 5J**). Compared to the control group, both *CLEC7A* and *RGS1* exhibited significantly increased expression in the ASCVD group (*P* < 0.0001). These findings indicate that *CLEC7A* holds greater diagnostic value in aspirin-resistant atherosclerosis (**Figure 5K**).

### 3.6 Immune infiltration of CLEC7A

**Figure 6:**
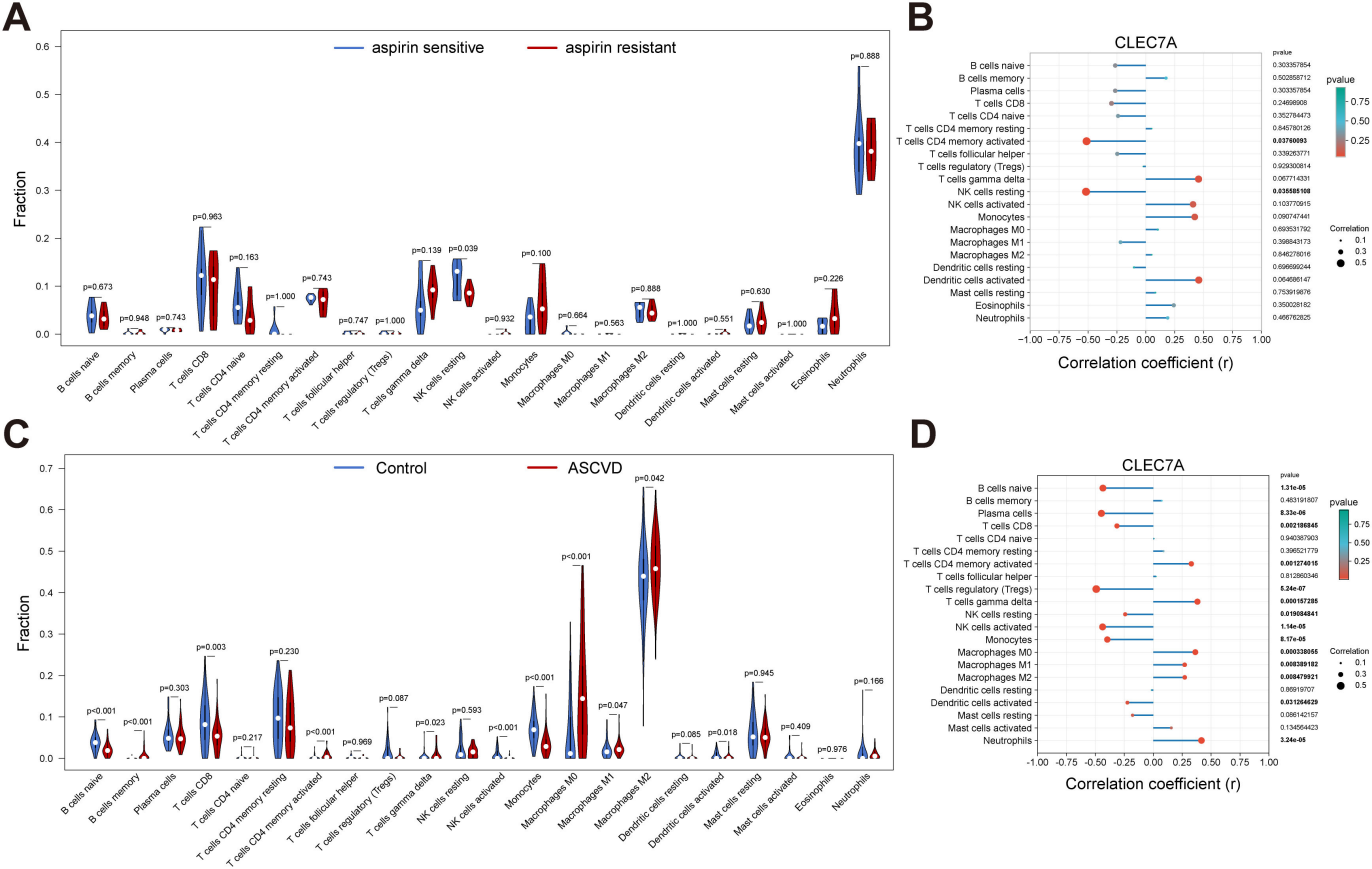
AR and ASCVD immune cell composition. **(A)** A violin diagram indicating that the AR group exhibited a significantly different immune cell composition. **(B)** Correlation between *CLEC7A* expression and immune cells in the AR group. **(C)** The violin diagram of the ASCVD group shows an evident difference in immune cell types. **(D)** Relationship between *CLEC7A* expression and immune cells in the ASCVD group. AR, Aspirin resistant; ASCVD, Atherosclerotic cardiovascular disease.

According to existing research, CLEC7A (C-type lectin domain family 7 member A) is associated with cardiovascular disease primarily through its indirect influence on the progression of atherosclerosis by regulating immune-inflammatory responses (23). CIBERSORT analysis was used to assess the abundance of different immune cell populations. Compared to the AS group, resting natural killer (NK) cells were significantly reduced in the AR group (**Figure 6A**). In ASCVD samples, memory B cells, activated memory CD4 T cells, T cells gamma delta, M0 macrophages, M1 macrophages, M2 macrophages, and activated dendritic cells were significantly increased, while naïve B cells, CD8 T cells, activated NK cells, and monocytes were significantly reduced (**Figure 6C**).

Additionally, the relationship between biomarkers and immune cell contents was investigated. In AR samples, activated memory CD4 T cells and resting NK cells were significantly negatively correlated with CLEC7A (**Figure 6B**). In ASCVD samples, CLEC7A was significantly negatively correlated with regulatory T cells (Tregs), plasma cells, activated NK cells, naïve B cells, monocytes, CD8 T cells, resting NK cells, and activated dendritic cells. In contrast, neutrophils, gamma delta T cells, M0 macrophages, activated memory CD4 T cells, M1 macrophages, and M2 macrophages were significantly positively correlated with CLEC7A (**Figure 6D**). Immune function appears to be critical for the development of aspirin non-responsiveness and ASCVD.

### 3.7 Single-gene GSEA and molecular regulatory network of CLEC7A

**Figure 7:**
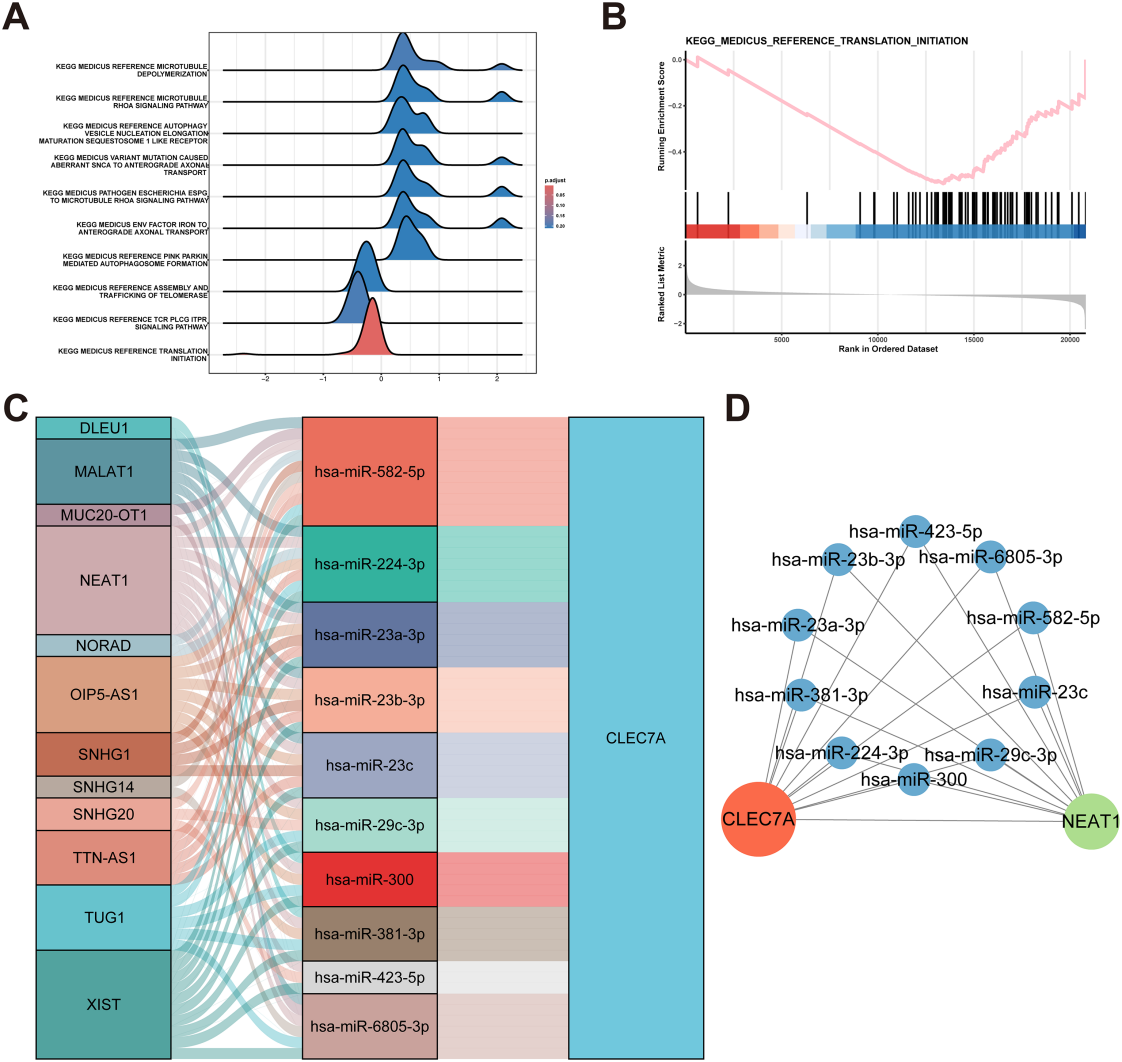
The single-gene GSEA and molecular regulatory networks of *CLEC7A*. **(A, B)** GSEA analysis of *CLEC7A*. **(C)** The alluvial diagram of the mRNA–miRNA–lncRNA network. **(D)** Circular regulatory signaling pathway. GSEA, Gene set enrichment analysis.

To identify the molecular pathways associated with CLEC7A in ASCVD, we performed GSEA analysis. This revealed that CLEC7A expression is most significantly linked to the “translation initiation” signaling pathway (**Figures 7A, B**), a process fundamental to protein synthesis in inflammatory cells. Subsequently, to uncover the upstream non-coding RNA regulators of CLEC7A, we constructed a comprehensive competing endogenous RNA (ceRNA) network. This analysis identified a complex regulatory landscape centered on CLEC7A, comprising 59 mRNA–miRNA–lncRNA axes (**Figure 7C**). Among these, several key axes prominently featured the lncRNA NEAT1 (e.g., CLEC7A\hsa-miR-582-5p\NEAT1 and CLEC7A\hsa-miR-224-3p\NEAT1). Furthermore, our network analysis identified 10 complex regulatory loops where lncRNAs could create feedback circuits, adding another layer of regulatory complexity (**Figure 7D**).

### 3.8 Verification of the ASCVD model and elevated CLEC7A expression in mice

**Figure 8:**
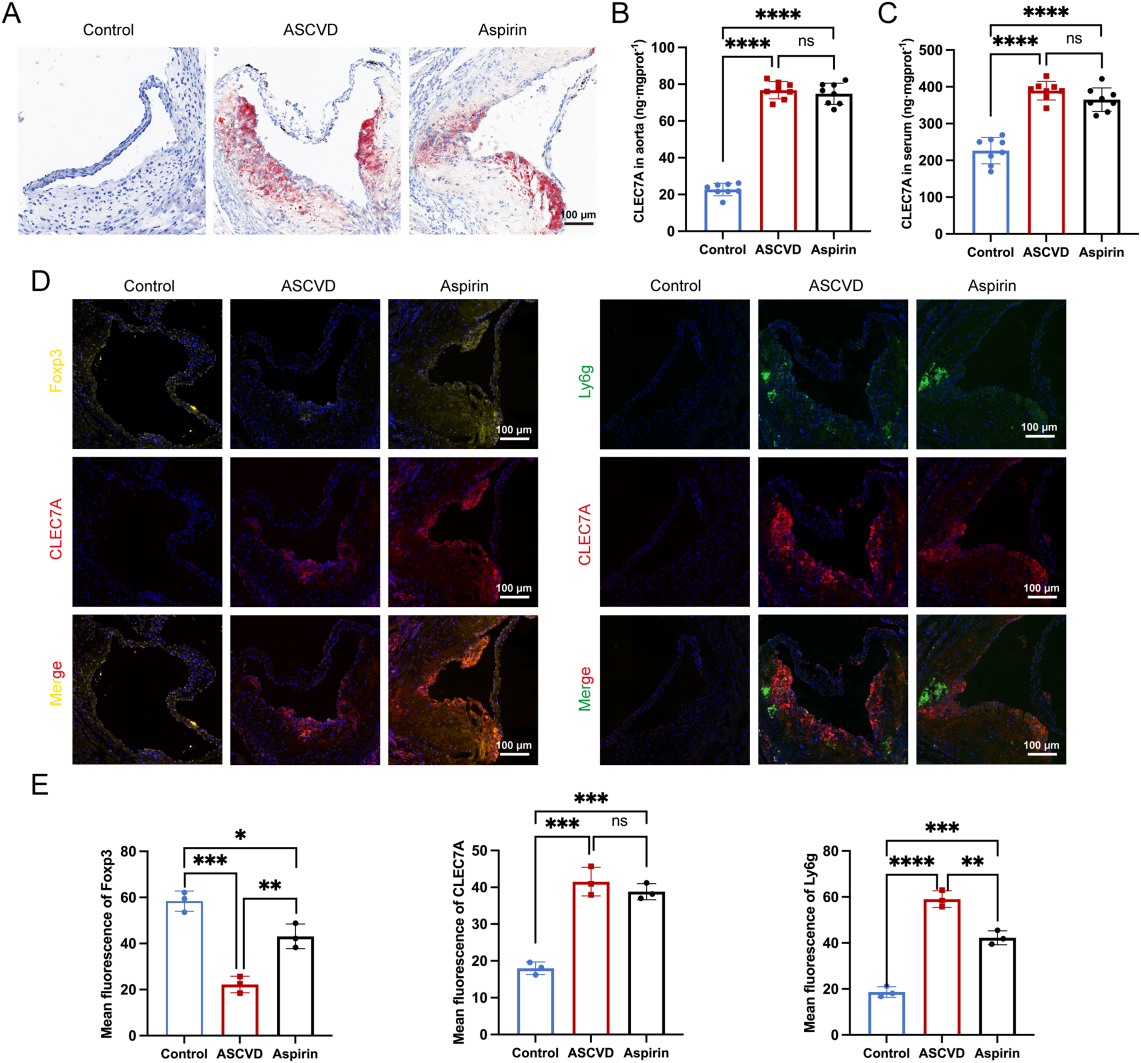
Validation of CLEC7A expression in mice. **(A)** Oily red staining of aortic tissue from mice, *n* = 3, bar = 100 μm. **(B, C)** Expression levels of CLEC7A in aortic tissue and serum from mice, *n* = 8. **(D)** Dual immunofluorescence staining of the T cell marker Foxp3 and neutrophil marker Ly6g with CLEC7A in mouse heart aortic valve tissue. **(E)** Immunofluorescence quantitative analysis, *n* = 3, bar = 100 μm. Data were shown as mean ± SD (^ns^*P*>0.05, ^*^P < 0.05, ^**^P < 0.01, ^***^P < 0.001, ^****^P < 0.0001).

In the control group, the aortic valve tissue exhibited an orderly structure with minimal folds or artifacts, and no red lipid staining was observed. In contrast, the ASCVD group showed marked tissue disruption with blurred architecture, prominent folds, and increased lipid plaque deposition, as evidenced by strong red staining, confirming successful model establishment (**Figure 8A**). Long-term aspirin treatment did not significantly reduce lipid plaque deposition compared with the ASCVD group (*P*>0.05). ELISA was subsequently performed on aortic tissue and serum samples to validate the expression level of the diagnostic biomarker CLEC7A. Consistent with the bioinformatic analysis, CLEC7A expression was significantly unregulated in both aortic tissue and serum of ASCVD mice compared with controls (*P* < 0.0001). No significant difference in CLEC7A expression was observed between the aspirin-treated group and the ASCVD group (*P*>0.05) (**Figures 8B, C**).

Double immunofluorescence staining demonstrated significant spatial association of CLEC7A with the regulatory T cell marker Foxp3 and neutrophil marker Ly6g in the aortic valve tissue (**Figure 8D**). Compared with controls, ASCVD mice showed a significantly lower mean fluorescence intensity of Foxp3^+^ regulatory T cells (*P* < 0.001) and a significantly higher mean fluorescence intensity of Ly6g^+^ neutrophils (*P* < 0.0001). Notably, the mean fluorescence intensity of CLEC7A was significantly increased in ASCVD mice (*P* < 0.001). However, although aspirin treatment could improve the mean fluorescence intensity of Foxp3^+^ and Ly6g^+^ (*P* < 0.01), it could not reverse the CLEC7A (*P*>0.05) (**Figure 8E**).

### 3.9 Virtual screening and CLEC7A-molecule interaction analysis

**Figure 9:**
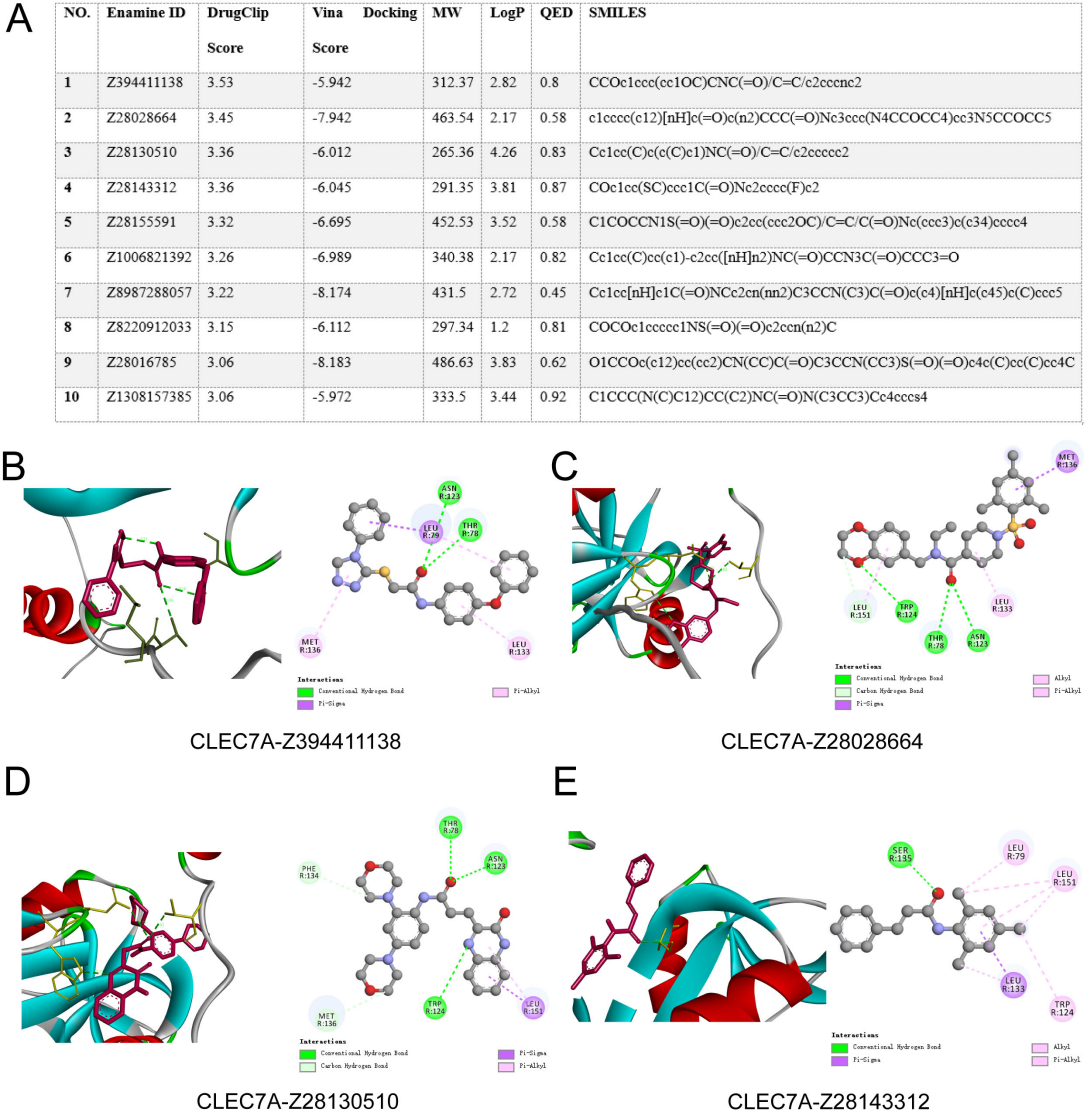
Virtual screening and CLEC7A-molecule interaction analysis. **(A)** Specific information on small molecules with DrugCLIP score >3 and Vina docking score <–5. **(B–E)** The docking conformations of the top four small molecules (Z394411138, Z28028664, Z28130510, Z28143312) with the CLEC7A protein.

The DrugCLIP platform was used to screen approximately 48 billion small molecules in the Enamine REAL library. Subsequently, the top 100 small molecules were selected from the Enamine library for Vina Dock re-docking. Finally, small molecules with DrugCLIP score >3 and Vina Docking score <–5 were retained (**Figure 9A**), and the docking conformations of the top four small molecules with CLEC7A protein were visualized (**Figures 9B–E**).

## 4 Discussion

The anticipated success of statins and other preventive measures in the late 20th century was expected to curb the ASCVD epidemic (24). However, rather than diminishing globally, the burden of this disease has surged to become the primary cause of global morbidity, years of life lost, and mortality (25). Despite optimal treatment with contemporary interventions and medications, the recurrence rate within the initial 12 months remains approximately 10% (26–28). Consequently, atherosclerosis persists as a significant clinical and public health challenge worldwide. Aspirin, the most commonly prescribed antiplatelet drug in clinical practice, has been the preferred medication in authoritative guidelines worldwide since its efficacy in patients with cardiovascular disease was established in the 1980s (29). Despite facing challenges over time, its fundamental role in secondary prevention of cardiovascular diseases remains unwavering. However, individuals with AR often do not receive adequate antithrombotic protection from aspirin, leading to adverse cardiovascular outcomes and side effects (30). A study found that among 293 patients with cardiovascular disease, 28% (810 cases) had AR. Data indicated that the incidence of cardiovascular events in resistant patients (39%) was significantly higher than in sensitive patients (16%) (31), underscoring the heightened risk of cardiovascular diseases associated with aspirin non-responsiveness. Another study demonstrated that aspirin non-responsiveness could triple the risk of major adverse cardiovascular events (32). Hence, early identification of individuals with aspirin non-responsiveness to evaluate the necessity for combination medication or alternative therapy before initiating antiplatelet treatment holds paramount clinical significance in the prevention and management of cardiovascular and cerebrovascular diseases.

Our study initially identified DEGs associated with aspirin non-responsiveness in ASCVD. Subsequently, we employed WGCNA to pinpoint modules associated with aspirin non-responsiveness in ASCVD. We then identified 17 hub genes using various algorithms. Using machine learning, we identified two key genes, *CLEC7A* and *RGS1*, both of which performed well in the ROC curve analysis. Notably, CLEC7A was significantly overexpressed in both the aspirin non-responsiveness and ASCVD groups (*P* < 0.0001), suggesting greater diagnostic value for aspirin-resistant atherosclerosis. Historically, the diagnosis of aspirin non-responsiveness has focused on platelet function testing, which poses challenges for targeted therapeutic interventions (33). Our multi-omics approach, integrating gene-level machine learning, identifies key protein factors, providing a foundation for potential therapeutic targets. Importantly, this study is the first to locate *CLEC7A* as significantly expressed in both ASCVD and aspirin non-responsiveness, with ROC analysis confirming its diagnostic potential. These findings suggest that *CLEC7A* not only predicts aspirin treatment efficacy but may also play a critical role in atherosclerosis pathogenesis.

We investigated CLEC7A, a C-type lectin receptor (CLR) predominantly expressed on myeloid cells and lymphocytes, which plays a multifaceted role in immunity (34–38). To explore its potential involvement in ASCVD and aspirin responsiveness, we first analyzed its relationship with immune cell populations. Our initial analysis revealed a significant negative correlation between CLEC7A and resting NK cells in the context of aspirin non-responsiveness, suggesting a potential link between CLEC7A, NK cell activity, and aspirin efficacy. However, a more comprehensive comparison between the control and ASCVD groups uncovered even stronger and potentially more central correlations. Specifically, CLEC7A expression was significantly associated with regulatory T cells (Tregs) and neutrophils, key orchestrators of inflammation in atherosclerosis. To validate these correlative findings and pinpoint the cellular localization, we performed double immunofluorescence staining on aortic valve tissues. The results visually confirmed a significant spatial association of CLEC7A with the Treg marker Foxp3 and the neutrophil marker Ly6g. Furthermore, our quantification of these cells in the disease context revealed that compared to the control group, ASCVD mice exhibited a decrease in Foxp3^+^ Tregs and an increase in Ly6g^+^ neutrophils. Critically, the expression pattern of CLEC7A was inversely correlated with this cellular shift. Importantly, aspirin treatment failed to reverse these CLEC7A expression changes or the altered Treg/neutrophil balance. This finding provides a potential association-based explanation for aspirin non-responsiveness. Taken together, these findings suggest that CLEC7A is not merely a marker but may be actively associated with ASCVD progression. We propose a hypothesis that CLEC7A contributes to aspirin non-responsiveness by being linked to the suppression of anti-inflammatory Tregs and the promotion of pro-inflammatory neutrophils. While our data provide strong correlative support for this model, further functional experiments are required to definitively validate this proposed mechanistic pathway.

Our findings suggest a clear, two-part mechanism linking CLEC7A to ASCVD progression and aspirin non-responsiveness. First, we predicted a potential ceRNA regulatory network where the pro-inflammatory molecule NEAT1 (39) inhibits miRNAs (hsa-miR-582-5p and hsa-miR-224-3p). This releases their suppression on CLEC7A, causing its overexpression. Second, our GSEA analysis showed that this elevated CLEC7A strongly activates the “translation initiation” pathway. This results in the mass production of inflammatory proteins, fueling plaque growth (40). However, its functional significance requires further experimental study. Critically, this heightened inflammation can activate platelets through pathways that aspirin cannot block, providing a direct molecular explanation for aspirin non-responsiveness. In essence, the NEAT1/CLEC7A axis creates a state of inflammation that both drives disease and blunts treatment.

We established atherosclerotic mouse models to compare CLEC7A expression in serum and aortic cardiac tissue between normal and atherosclerotic mice. CLEC7A was significantly overexpressed in both serum and cardiac aortic tissues of atherosclerotic mice. However, long-term aspirin treatment cannot reduce its expression. Virtual screening subsequently identified potential small-molecule candidate compounds (Z394411138, Z28028664, Z28130510, Z28143312) targeting the key gene CLEC7A.

Our study does have limitations. We have yet to recruit a sufficient number of patients experiencing aspirin treatment failure to validate key genes clinically, though this remains a focus of our efforts. Furthermore, experimental validation of the identified small-molecule drugs is necessary.

## 5 Conclusions

Our bioinformatics analysis has identified two key genes and developed a predictive model for suboptimal aspirin response in ASCVD. Notably, CLEC7A emerges as a potential ASCVD risk factor and may influence immune cell involvement in aspirin efficacy. Additionally, we have investigated the molecular regulatory network of CLEC7A and its implications for targeted therapy. These findings can stratify patients by response likelihood and guide tailored antiplatelet therapy to enhance the efficacy of ASCVD prevention and treatment.

## Data Availability

The original contributions presented in the study are included in the article/**Supplementary Material**. Further inquiries can be directed to the corresponding author/s.
