# Supplementary material for: CLEC7A as a potential biomarker of atherosclerotic response to aspirin therapy: a multi-omics analysis
Source: Front Immunol. 2026 Jul 29;17:1727020. doi: 10.3389/fimmu.2026.1727020 (PMC13461398; doi:10.3389/fimmu.2026.1727020)
Supplement: Supplementary file 1 [file Table1.docx]

***Supplementary Material***

**Supplementary Table S1. GO enrichment analysis.**

| ID | Description | classification | pvalue | all | differ gene |
| --- | --- | --- | --- | --- | --- |
| GO:1990830 | cellular response to leukemia inhibitory factor | Biological Process | 0.00030821 | 94 | 5 |
| GO:1990823 | response to leukemia inhibitory factor | Biological Process | 0.00032372 | 95 | 5 |
| GO:0032613 | interleukin-10 production | Biological Process | 0.00061097 | 62 | 4 |
| GO:0032653 | regulation of interleukin-10 production | Biological Process | 0.00061097 | 62 | 4 |
| GO:0045428 | regulation of nitric oxide biosynthetic process | Biological Process | 0.00061097 | 62 | 4 |
| GO:0080164 | regulation of nitric oxide metabolic process | Biological Process | 0.00068925 | 64 | 4 |
| GO:0006809 | nitric oxide biosynthetic process | Biological Process | 0.00131404 | 76 | 4 |
| GO:0070373 | negative regulation of ERK1 and ERK2 cascade | Biological Process | 0.00144706 | 78 | 4 |
| GO:0046209 | nitric oxide metabolic process | Biological Process | 0.00166378 | 81 | 4 |
| GO:0002765 | immune response-inhibiting signal transduction | Biological Process | 0.00168615 | 10 | 2 |
| GO:0051010 | microtubule plus-end binding | Molecular Function | 0.00035684 | 21 | 3 |
| GO:0004726 | non-membrane spanning protein tyrosine phosphatase activity | Molecular Function | 0.00193246 | 10 | 2 |
| GO:0031690 | adrenergic receptor binding | Molecular Function | 0.00781013 | 20 | 2 |
| GO:0035014 | phosphatidylinositol 3-kinase regulator activity | Molecular Function | 0.00781013 | 20 | 2 |
| GO:0001965 | G-protein alpha-subunit binding | Molecular Function | 0.01114922 | 24 | 2 |
| GO:0030247 | polysaccharide binding | Molecular Function | 0.01206609 | 25 | 2 |
| GO:0019207 | kinase regulator activity | Molecular Function | 0.02033876 | 233 | 5 |
| GO:0051117 | ATPase binding | Molecular Function | 0.02192864 | 89 | 3 |
| GO:0060090 | molecular adaptor activity | Molecular Function | 0.03791247 | 369 | 6 |
| GO:0008234 | cysteine-type peptidase activity | Molecular Function | 0.03896593 | 190 | 4 |
| GO:0030990 | intraciliary transport particle | Cellular Component | 0.00042567 | 24 | 3 |
| GO:0072686 | mitotic spindle | Cellular Component | 0.0005007 | 161 | 6 |
| GO:0099738 | cell cortex region | Cellular Component | 0.0025456 | 44 | 3 |
| GO:0097542 | ciliary tip | Cellular Component | 0.00307499 | 47 | 3 |
| GO:0030992 | intraciliary transport particle B | Cellular Component | 0.00486217 | 17 | 2 |
| GO:0005876 | spindle microtubule | Cellular Component | 0.01011461 | 72 | 3 |
| GO:0036064 | ciliary basal body | Cellular Component | 0.01789821 | 161 | 4 |
| GO:0009898 | cytoplasmic side of plasma membrane | Cellular Component | 0.02220257 | 172 | 4 |
| GO:0032391 | photoreceptor connecting cilium | Cellular Component | 0.02662295 | 41 | 2 |
| GO:0099568 | cytoplasmic region | Cellular Component | 0.02676294 | 271 | 5 |

**Supplementary Table S2. KEGG enrichment analysis.**

| Description | GeneRatio | pvalue | geneID | Count |
| --- | --- | --- | --- | --- |
| Thyroid hormone synthesis | 0.057692308 | 0.008157 | ATP1B1/ADCY9/CREB5 | 3 |
| Insulin secretion | 0.057692308 | 0.011849 | ATP1B1/ADCY9/CREB5 | 3 |
| cGMP-PKG signaling pathway | 0.076923077 | 0.013171 | ATP1B1/ADRA2A/ADCY9/CREB5 | 4 |
| Aldosterone synthesis and secretion | 0.057692308 | 0.016813 | ATP1B1/ADCY9/CREB5 | 3 |
| Alanine, aspartate and glutamate metabolism | 0.038461538 | 0.017681 | ASRGL1/GFPT2 | 2 |
| Fatty acid degradation | 0.038461538 | 0.023475 | CYP2U1/ACAT2 | 2 |
| Vasopressin-regulated water reabsorption | 0.038461538 | 0.024506 | ADCY9/CREB5 | 2 |
| TNF signaling pathway | 0.057692308 | 0.027924 | CASP7/CREB5/CXCL10 | 3 |
| Growth hormone synthesis, secretion and action | 0.057692308 | 0.029765 | ADCY9/SOCS2/CREB5 | 3 |
| Endocrine and other factor-regulated calcium reabsorption | 0.038461538 | 0.034593 | ATP1B1/ADCY9 | 2 |
